# Supplementary material for: Learning curve and surgical outcome of robotic assisted colorectal surgery with ERAS program
Source: Sci Rep. 2022 Nov 29;12:20566. doi: 10.1038/s41598-022-24665-w (PMC9709162; doi:10.1038/s41598-022-24665-w)
Supplement: Supplementary file 2 — Supplementary Information 2. [file 41598_2022_24665_MOESM2_ESM.zip › RAL Raw data-Ñ[▒K/093/OP N_ñw╝╨░O▒Kñσ.pdf]

W52 / W52

臺中榮民總醫院
手術室護理記錄

[redacted]

基本資料

- 室別： 開刀房-17室，手術科別： CRS，手術類別： 常規，日期： 2021/06/26
- 進入時間： 0800 ； 麻醉開始時間： 0810 ； Time Out時間： 0925 ； 劃刀時間： 0926 ； 手術結束時間： 1315 送出時間： 1325 ；
- 麻醉方式： GE
  診斷： S COLON CA 20CM FAV
  手術名稱： Robot Assisted Colectomy(L)
  手術局部用藥： 2%Xylocaine jelly
  術前預防性抗生素：
  - 抗生素名稱一： Veterin 1GM IVD由麻醉護理師給予
  - 時間： 0920
  - 上次簽章: 陳妍希

  ，追加預防性抗生素：
- 手術醫師： 蔣鋒帆，陳映菁，林佑儒
- 麻醉醫師： VS 李富榮
-
  - 刷手護士A ： 黃鈺雯，時間： 0800，刷手護士B ： 何佳青，時間： 0800，接替刷手一： 陳妍希，時間： 1230，接替刷手二： 黃鈺雯，時間： 1300
  - 流動護士A ： 陳妍希，時間： 0800，接替流動一： 何佳青，時間： 1030，接替流動二： 陳妍希，時間： 1200
- 更多接替及流動護士： 接替流動三： 何宛臻，時間： 1230

皮膚狀況

A-V Shunt： 無
術前： 完整
  記錄者： 上次簽章: 陳妍希
術後： 完整
  記錄者： 何宛臻

手術準備

手術臥式 單- -手術臥式

-
  - 手術臥式： 截石臥式
  - 使用附件： 溫毯 38 ℃，頭圈，腳架，其它： silicon padX2、海綿
  - 皮膚準備： 10%Aq Betadine，Hibiscrub，2% Chlorhexidine
  - 電燒： 使用電燒: 電極片位置： 其它： 迴路電極墊，雙極電燒，記錄者： 上次簽章: 陳妍希

止血帶T： 無

植入物

- 植入物名稱/號碼/數量/部位： Davinci EndoWrist Stapler 45-4.3mmx2/S colon

，記錄者： 何宛臻

標本送檢

- 細菌培養 無
- 細胞學 無
- 冰凍切片 無
- 病理標本
  組織名稱一： s colon
  ，
  組織名稱二： distal cutend
  ，
  組織名稱三： IMA root

- 截肢 無

標本交付 標本： 3 袋，標本： 0 盒，標本： 0 瓶，細胞學： 0 管，Culture Tube： 0 管，被交付醫師： CR 陳映菁，

- 記錄者： 何宛臻
- 時間： 1320

傷口術後數料

傷口數料：　　　　傷口數料： 無

- 請於圖上標示部位：

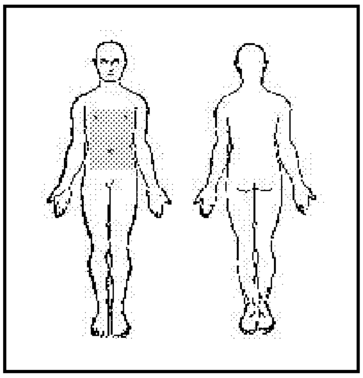

- 傷口數料： manport

導管

導管	天數	導管資訊	插入日期	插入時間	更換日期	拔除日期	拔除時間	拔除原因	預計拔除/更換	預計拔除/更換日期
Foley(1)	1	號碼:16 Fr.; 部位:膀胱;	2021/06/26	0840						
Double Lumen CVC(1)	1	號碼:8 Fr.; 深度:15 cm; 部位:jugular (右); 象限:RU;	2021/06/26	0855						
A-line (1)	1	號碼:20 G; 部位:右手;	2021/06/26	0845						

用物計數

- 手術中出血量： minimal c.c.
- 手術中尿量： 500 c.c.
-
    - 其它： 無

用物計數： 多切口同時縫合

- 1.器械： 正確，2.縫針： 正確，3.刀片： 正確，4.紗布： 正確，5.腹部墊： 未使用，6.紗球： 未使用，7.PEANUTS ： 未使用，8.紗條： 未使用，9.絲帶： 未使用，10.針頭： 未使用，11.Vessel loop： 未使用，12.Boots： 未使用，13.Jelco： 未使用，14.棉條： 未使用，
    - 第一次：
        - 刷手護士 ： 黃鈺雯
        - 流動護士： 何宛臻
        - 時間： 1210

    ,
    - 第二次：
        - 刷手護士 ： 黃鈺雯
        - 流動護士： 何宛臻
        - 時間： 1225

    ,
    - 第三次：
        - 刷手護士 ： 黃鈺雯
        - 流動護士： 何宛臻
        - 時間： 1300

備註

備註： davinci 30度 XI scopy:No.2

- 記錄者： 上次簽章: 陳妍希
- 時間： 0805

填寫護理人員 何宛臻

正式摘要

W52 / W52

doctorRank:B
